# Supplementary material for: Potential Anti-Obesity, Anti-Steatosis, and Anti-Inflammatory Properties of Extracts from the Microalgae Chlorella vulgaris and Chlorococcum amblystomatis under Different Growth Conditions
Source: Mar Drugs. 2021 Dec 22;20(1):9. doi: 10.3390/md20010009 (PMC8781425; doi:10.3390/md20010009)
Supplement: Supplementary file 1 [file marinedrugs-20-00009-s001.zip › Suplementary Figures.pdf]

marine drugs

MDPI

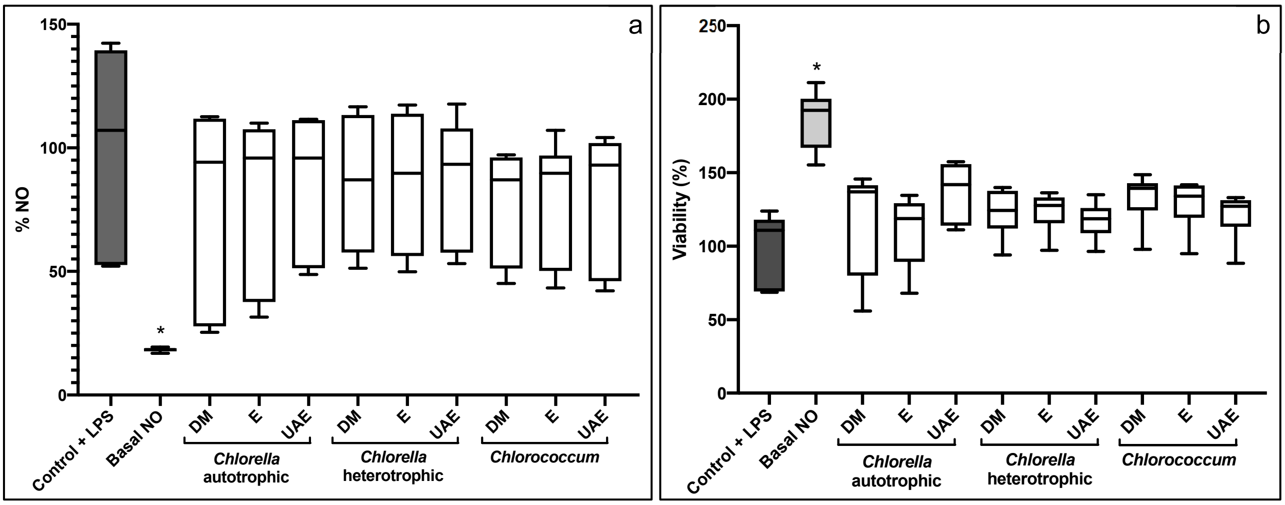

Figure S1. Anti-inflammatory and viability assays in RAW264.7 cell line exposed to 10 µg/ml of extracts. Dark grey represents inflammation control (induced by LPS and containing the same DMSO content as extracts); light grey represents basal NO without induction of inflammation by LPS. (a) results for the anti-inflammatory assay; (b) results for cell viability. Solvents used for preparation of the extracts: DM: dichloromethane-methanol (2:1); E: ethanol; UAE: ultrasound-assisted extraction with ethanol. The data presented is derived from three independent assays in duplicates (n = 6) and shown as box-and-whisker plots (5–95 percentiles). Statistical differences compared to DMSO control are indicated by asterisks. (p-value < 0.05).

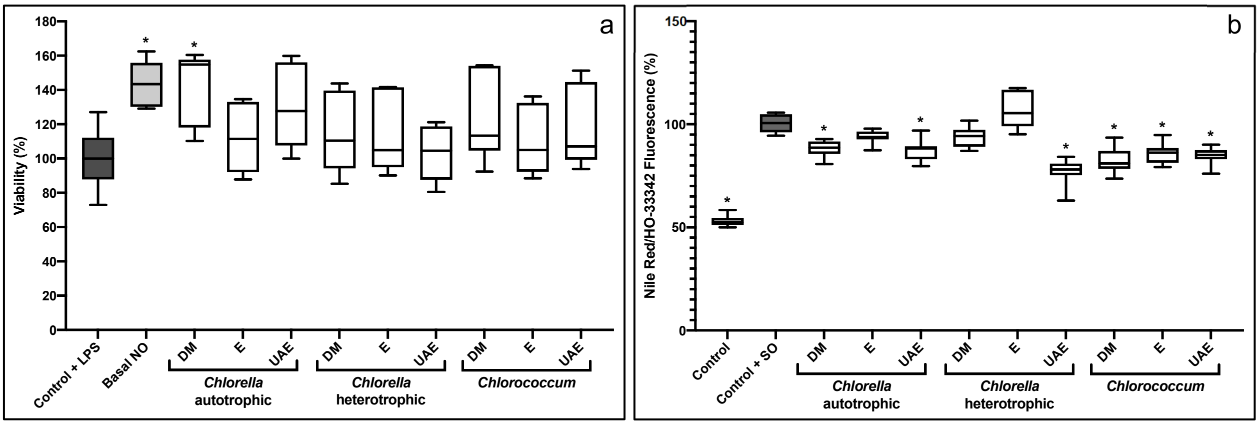

Figure S2. Anti-steatosis activity assay in fatty acid overloaded HepG2 cells and cell viability by SrB method, at 10 µg/ml extract. (a) Nile red and HO-33342 fluorescence quantification ratio expressed as percentage compared to fat-overloaded control (Control + SO); (b) HepG2 cell viability using SrB method. Light grey represents DMSO control; dark grey represents control + SO. Solvents used for preparation of the extracts: DM: dichloromethane-methanol (2:1); E: ethanol; UAE: ultrasound assisted extraction with ethanol. Data were derived from two independent assays in triplicates (n = 6) and shown as box-and-whisker plots (5–95 percentiles). Statistical differences compared to DMSO+SO control is indicated by asterisks (p < 0.0001).
